# The functional significance of delta oscillations in cognitive processing

**DOI:** 10.3389/fnint.2013.00083

**Published:** 2013-12-05

**Authors:** Thalía Harmony

**Affiliations:** Departamento de Neurobiología Conductual y Cognitiva, Instituto de Neurobiología, Universidad Nacional Autónoma de MéxicoQuerétaro, México

**Keywords:** EEG delta, attention, working memory, frontal lobes, inhibition, cognition

## Abstract

Ample evidence suggests that electroencephalographic (EEG) oscillatory activity is linked to a broad variety of perceptual, sensorimotor, and cognitive operations. However, few studies have investigated the delta band (0.5–3.5 Hz) during different cognitive processes. The aim of this review is to present data and propose the hypothesis that sustained delta oscillations inhibit interferences that may affect the performance of mental tasks, possibly by modulating the activity of those networks that should be inactive to accomplish the task. It is clear that two functionally distinct and potentially competing brain networks can be broadly distinguished by their contrasting roles in attention to the external world vs. the internally directed mentation or concentration. During concentration, EEG delta (1–3.5 Hz) activity increases mainly in frontal leads in different tasks: mental calculation, semantic tasks, and the Sternberg paradigm. This last task is considered a working memory task, but in neural, as well as phenomenological, terms, working memory can be best understood as attention focused on an internal representation. In the Sternberg task, increases in power in the frequencies from 1 to 3.90 Hz in frontal regions are reported. In a Go/No-Go task, power increases at 1 Hz in both conditions were observed during 100–300 ms in central, parietal and temporal regions. However, in the No-Go condition, power increases were also observed in frontal regions, suggesting its participation in the inhibition of the motor response. Increases in delta power were also reported during semantic tasks in children. In conclusion, the results suggest that power increases of delta frequencies during mental tasks are associated with functional cortical deafferentation, or inhibition of the sensory afferences that interfere with internal concentration. These inhibitory oscillations would modulate the activity of those networks that should be inactive to accomplish the task.

## Introduction

For several decades, electroencephalographic (EEG) recordings have been used for the study of cognitive functions. These oscillations have been classified by their frequency since the very early papers after the discovery of EEG by Berger (Adrian and Mathews, 1934). The technological development of digital EEG resulted in a more precise quantification of EEG frequencies and the definition of bands of frequencies that have the approximate following ranges: 0.5–3.5 Hz for delta, 4–7.5 Hz for theta, 8–12.5 Hz for alpha, 13–30 Hz for beta and higher than 30 Hz for gamma. A very slow activity, lower than 1 Hz oscillations, has also been considered (Steriade et al., 1993).

At the moment, a large body of evidence suggests that oscillatory activity in all frequency bands is linked to a broad variety of perceptual, sensorimotor, and cognitive operations (Basar, 1998; Aoki et al., 1999; Klimesch, 1999; Palva and Palva, 2007). However, in recent years, studies relating EEG oscillations with cognitive processes have focused on alpha and theta frequencies, beta and gamma frequencies and, more recently, very slow activity.

Only few actual references have studied the delta band during different cognitive processes. According to Knyazev (2012), functional delta oscillations appear to be implicated in the synchronization of brain activity with autonomic functions, in motivational processes associated with both reward and atavistic defensive mechanisms, in higher emotional involvement, and in cognitive processes related to attention and the detection of motivationally salient stimuli in the environment (Knyazev, 2007; Knyazev et al., 2009). Delta has also been related to behavioral inhibition (Kamarajan et al., 2004; Knyazev, 2007; Putman, 2011).

In sum, several cognitive neuroscientists consider delta oscillations to be involved in many cognitive processes (Basar et al., 2001).

Delta oscillations were also studied in the 1970s and 1980s. Vogel et al. (1968) reported a high correlation between the number of slow waves in the EEG during the performance of a task and the proficiency in the execution of the task. To explain the apparent contradiction between the increase of delta waves during mental tasks and the fact that this activity is the main characteristic of slow wave sleep, the authors postulated the existence of a type of inhibition that would selectively suppress inappropriate or non-relevant neural activity during the performance of a mental task.

EEG delta during the performance of mental tasks has not been studied intensively in the general literature for approximately 30 years. The current review is focused on delta oscillations to promote interest in this research topic and to increase the knowledge of a more general audience in relation to EEG oscillations and cognition. Readers interested in this area may consult different reviews in relation to theta (Klimesch, 1999; Bastiaansen and Hagoort, 2003; Babiloni et al., 2006; Osipova et al., 2006; Klimesch, 2012), alpha (Klimesch et al., 2007), beta (Pfurtscheller et al., 2005) and gamma activity (Jensen et al., 2007).

Following Vogel’s idea, my colleagues and I performed several experiments that showed increases in delta activity during the performance of different mental tasks. These findings will be described in the Experimental Data section, together with interesting papers relating meditation to delta. However, previous to this discussion, it is necessary to give a summarized review of the origin of delta waves, the basic aspects of electromagnetic oscillations, and their role in the modulation of cerebral networks. Knowledge of these issues is crucial to understand the main hypothesis postulated in relation to the functional role of EEG oscillations and to understand the interpretation of results described in Experimental Data. A summarized review of the attention networks has also been included because many interpretations for the increases in delta power have been related to the activation of one type of network and the inhibition of the other.

Our goal in this review is to present data and propose the hypothesis that sustained delta oscillations inhibited interferences that may affect the performance of the task, possibly by modulating the activity of those networks that should be inactive to accomplish the task.

## The Basic aspects of electromagnetic oscillations and their role in the modulation of cerebral networks. (For a review, see Buzsáki et al. (2012))

In 1991, Lopes da Silva (1991) proposed in a seminal paper that an oscillation in neural networks was not simply a by-product of the activity of neuronal networks but may have a functional significance in brain function. He examined the fact that brain rhythms may have functional implications for the working of neural networks in relation to two cases: the possibility that oscillations may subserve a gating function and that those oscillations may play a role in the formation of assemblies of neurons that represent given stimulus patterns.

Each oscillatory cycle is a temporal processing window, signaling the beginning and termination of the encoded or transferred messages. The wave length of the oscillatory category determines the temporal windows of processing and, indirectly, the size of the neuronal pool involved. From this speculation, it follows that different frequencies favor different types of connections and different levels of computation. In general, slow oscillators can involve many neurons in large brain areas, whereas the short time windows of fast oscillators facilitate local integration, largely due to the limitations of axon conduction delays. Computation in the brain means that information is moved from one place to another. The path length of network connectivity is very critical in this process. Because the synaptic path and effective connectivity determine the possible routes for shuttling information from structure to structure, the cycle lengths (i.e., periods) of the oscillation limit how far information gets transferred in one step. Fast oscillations, therefore, favor local decisions, whereas the involvement of distant neuronal groups in distinct structures requires more time to obtain a global consensus.

To understand the recent proposals regarding the functional role of brain oscillations, the basic mechanisms that give rise to these oscillations will be summarized. In the brain, all excitable membranes, as well as all types of transmembrane currents, contribute to the extracellular field. This field is the superposition of all ionic processes, ranging from fast action potentials to the slowest fluctuations in glia. Thus, extracellular currents can emerge from multiple sources, with synaptic activity being the most important source. Na^+^ spikes were thought to not contribute substantially to the EEG; however, synchronous action potentials from many neurons can contribute to its high-frequency components. Long-lasting (10–100 ms) Ca^2+^-mediated spikes, currents flowing through hyperpolarization deinactivated cyclic nucleotide-gated channels (I_h_ currents) and low-threshold (hyperpolarization-induced) transient Ca^2+^ currents (I_T_ currents), spikes after hyperpolarizations, gap junctions, changes in the membrane potentials of the glia cells, and ephaptic effects have been considered as potential sources of the EEG.

According to Jefferys (1995), there are three types of electrical communication between neurons: (1) electronic coupling between specific neurons through specialized “gap junctions”; (2) “ephaptic” coupling between closely opposed, but noncontiguous, neuronal membranes; and (3) “field” interactions, in which the geometry of a structure promotes the generation of large extracellular fields that alter the excitability of neighboring neurons when suitably oriented. This author delimitated between the last two possibilities, considering “ephaptic” coupling as the close apposition of neuronal processes from two or a few neurons, as in the mammalian Purkinje cells. “Field” effects (or interactions) are considered the electrical communication that depends on large voltage fields generated by the synchronous activity of many neurons with a suitable (usually laminar) geometry. The most common function of electrical transmission is neuronal synchronization. In the words of Buzsáki et al. (2012), “the resistivity of the extracellular medium in the mammalian brain and the highly transient nature of spikes, make unlikely that spikes from individual neurons greatly affect the excitability of nearby neurons through ephaptic coupling. However, the situation is very different when many neurons are simultaneously active, as such synchrony can generate strong spatial gradients in the extracellular voltage”. These authors further stated “Experiments have shown that small-amplitude, slow-frequency application of extracranial currents (trans-cranial electrical stimulation) has a detectable effect on neuronal activity and cognitive function; the small but effective voltage gradients brought about in brain tissue by such external fields are comparable to the voltage gradients produced by population patterns in vivo under physiological conditions”. In slice preparations of the primary visual cortex of anesthetized ferrets, (Frölich and McCormick, 2010) demonstrated that fluctuations in the extracellular potentials produced by the structured neocortical neuronal activity directly influence network activity by modulating the neuronal membrane voltage; this phenomenon forms a feedback loop between the neuronal activity and the endogenous electric fields. This significant susceptibility of the active networks to the extracellular fields that only causes small changes in the membrane potential in individual neurons suggests that endogenous potentials could guide neocortical network activity (Buzsáki et al., 2012).

The important point is that cortical neuronal activity changed under the influence of fields as weak as 2.5 mV/mm, smaller than the spontaneous slow potentials generated by the brain under physiological conditions (Jefferys, 1995). Therefore, field effects likely have some role in the physiological EEG, although assessment of its relative importance compared with the synaptic activity that occurs at the same time awaits more detailed cellular and network analysis.

The chemical synapse remains the key to the interaction between neurons. However, nonsynaptic mechanisms play significant roles in neuronal function. In general, the electrical interactions, gap junctions, ephapses, and field effects can mediate neuronal synchrony on a fast, millisecond time scale. In contrast, ionic and other chemical mechanisms operate over much slower time scales (i.e., tens of milliseconds and upwards (Jefferys, 1995)).

In the generation of the EEG, although all neuron types contribute to the extracellular field, pyramidal cells generate strong dipoles along the somatodendritic axis due to their geometry. Such dipoles give rise to an open field as there is considerable spatial separation of the active sink (or the source) from the return currents. This separation induces substantial ionic flow in the extracellular medium. The two most important determinants of the extracellular field strength are the spatial alignment of the neurons and the temporal synchrony of the dipole moments they generate. This last factor, the temporally synchronous fluctuations of the membrane potential in large neuronal aggregates, is extremely important. Synchrony, which is often produced through network oscillations, explains why different brain states are associated with dramatically different magnitudes of local field potentials (Buzsáki et al., 2012).

### The origin of delta oscillations

During non-rapid-eye-movement sleep, the membrane potential of cortical neurons periodically shifts (0.5–1.5 Hz) between a hyperpolarized “down” state and a more depolarized “up” (that is, spiking) state. The largest amplitude up–down shifts of the membrane voltage occur in the fifth layer of the large pyramidal neurons; the large voltage shifts in the somata of the synchronously active–silent neurons have been proposed to induce the formation of an extracellular dipole between deep (infragranular) and superficial (supragranular) layers. At least part of the cessation of spiking during the down states can be explained by after hyperpolarizations of the synchronously bursting pyramidal cells in the up state. The temporally coordinated silent down state of nearby neurons is associated with a positive potential in the infragranular layers and a negative potential in the supragranular layers.

In an excellent large review (Knyazev, 2012), Knyazev quoted Murphy et al. (2009). These authors, using high-density EEG source modeling, showed that the slow waves during sleep (SWS), known as individual spontaneous slow waves (0.5–6 Hz), have distinct cortical origins, propagate uniquely across the cortex, and involve unique subsets of cortical structures. However, when the waves are examined en masse, diffuse hot spots of slow wave origins could be noted. These hot spots are centered at the left insula and the medial cingulate gyrus. Slow-wave propagation along the anterior posterior axis of the brain is largely mediated by a cingulate highway. The highest density of streamlines was in the anterior portions of the cingulate. As a group, slow waves are associated with large currents in the medial frontal gyrus, the middle frontal gyrus, the inferior frontal gyrus, the anterior cingulate, the precuneus, and the posterior cingulate. This topographic anatomical correspondence between the putative site of delta generation and the cortical terminal field of the mesotelencephalic dopamine system has been used as evidence for the hypothesis linking delta oscillations with motivation (Alper et al., 2006; Knyazev et al., 2009; Knyazev, 2012). Data supporting an increase of delta power in motivationally relevant states (see Knyazev, 2007; for a review) also support this hypothesis.

Studies correlating positron emission tomography (PET) and EEG indicate a positive correlation between waking delta and PET metabolism in the medial frontal cortex (Alper et al., 2006). This positive correlation contrasts with the negative relationship between the delta of SWS and PET metabolism (Maquet, 1997) or regional blood flow in the medial frontal cortex (Hofle et al., 1997). These apparently opposite relationships of delta to metabolism and blood flow in the medial frontal cortex in sleep vs. waking states have been used to argue the functional distinction between the waking and SWS delta (Murphy et al., 2009). However, Dang-Vu et al. (2008) discussed how source modeling and the correlation of EEG with PET and fMRI signals consistently show the location of both waking and SWS delta sources in the medial prefrontal, orbitofrontal, and anterior cingulate cortices.

In sum, “much evidence converges on showing that both waking and sleep delta waves mostly originate from the medial frontal cortical regions. Other hot spots include the insula, which is a higher association area for bodily signals (Mesulam and Mufson, 1982), the nucleus accumbens, and tegmental brainstem area” (Knyazev, 2012, p. 684). Unfortunately, most of these circuits are located deep in the brain, and their electrical activity is not directly accessible on the scalp. Although we may learn much from findings that come from fMRI, PET, and animal research, caution should be exercised when these findings are applied to the explanation of human EEG data.

The origin of delta waves during cognitive processes remains unknown. It has been hypothesized that low frequency oscillations of delta and theta ranges are associated with motivational and emotional processes (Knyazev, 2007). Considerable evidence confirms a link between theta activity and emotional states both in animals and in humans (Basar, 1998; Klimesch, 1999, 2012; Klimesch et al., 2007; Knyazev et al., 2009).

The role of delta waves during mental tasks has also been postulated to be associated with cortical deafferentation or with the inhibition of the sensory afferences that interfere with internal concentration (Fernández et al., 1995; Harmony et al., 1996).

Delta oscillations are a characteristic of sleep. From the standpoint of this review, sleep is an interesting natural model of functional decortications (Rial et al., 2007) when advanced operational brain systems are inactive. In this review, it is proposed that an alternative mechanism for ignoring inputs is through inhibition-based oscillations that can provide the prolonged periodic suppression of activity (Buzsáki, 2006). These inhibitory oscillations may modulate the activity of those networks that should be inactive to accomplish the task.

## The attention networks

In 1890, William James, in his definition of attention, distinguished two different aspects of this process: attention to external objects and attention to internal processes, such as “trains of thought”. The ability to willfully and persistently maintain that selective content in consciousness is what we understand to be the capacity to concentrate attention.

A major advance in the neurobiological bases of attention has been the recognition that separate neural systems mediate different aspects of attention (Posner and Petersen, 1990). Currently, two functionally distinct and potentially competing brain networks have been identified, which can be broadly distinguished by their contrasting roles in attention to the external world vs. the internally directed mentation involving long-term memory (Corbetta and Shulman, 2002). The dorsal attention system has properties consistent with the system supporting externally directed cognition (Fox et al., 2006).

The second system—the hippocampal-cortical memory system or the ventral system—is part of a network of regions that are active during passive mental states linked to internally directed cognition, including the recollection of the past and thinking about the future (often labeled the default network (Raichle et al., 2001; Buckner et al., 2008)). This brain system includes regions in the ventral medial prefrontal cortex, the posterior inferior parietal lobule, the retrosplenial cortex, the posterior cingulate, and the lateral temporal lobe.

Several studies have shown that spontaneous activity within the dorsal attention system and the ventral memory system is negatively correlated, indicating the segregation of competing processes. Regarding the dorsal attention and ventral memory systems, this segregation may arise from their divergent roles in processing information from the external world vs. internal mentation. This intriguing observation raises the question of how these two systems interact and whether there are control systems that either integrate information from the two systems or regulate their activity.

Vincent et al. (2008) described a third system: the frontoparietal control system, which includes many regions identified as supporting cognitive control and decision-making processes, including the lateral prefrontal cortex, the anterior cingulate cortex, and the inferior parietal lobule.

In contrast, Fuster (2008) established that “attention has two complementary components: an intensive, selective component, and an exclusionary one. The first, as it applies to the initiation and execution of action, seems based primarily in dorsolateral frontal cortex. The exclusionary component of attention, on the other hand, is the equivalent of the inhibitory control of interference, which seems primarily based in ventral frontal cortex. In any case, a ubiquitously active cognitive function, such as attention, cannot be localized in any particular brain structure. Selective executive attention can be construed as a property of frontal networks in operation. The focus of executive attention would shift from one domain of action to another as different networks or their parts excite one another in the perception–action cycle…”. Fuster further states “Interference control is the third executive-attention sub-function of the prefrontal cortex. It is the exclusionary or suppressive aspect of executive attention. This function protects behavioral structures from external or internal interference. Among the important sources of interference are the sensory or executive memories that are similar to those in current action but inappropriate to it. By suppressing distraction, executive attention is served. Interference control is an inhibitory function based primarily in orbitomedial prefrontal cortex and exerted from there on a variety of cortical and subcortical regions, prominently the basal ganglia” (Vincent et al., 2008).

This assertion is supported by several neuroimaging studies that have shown that any task requiring concentrated attention activates areas of the lateral and medial prefrontal cortex in addition to the posterior cortical areas of the perceptual specialization (Kosslyn, 1988; Pardo et al., 1991).

In this paper, we propose that the mental tasks requiring internal concentration, as it has been described above, or internal mentation, as it was recently named, are accompanied by the apparition of EEG delta oscillations, which inhibit the other processes that interfere with the resolution of the mental task.

## Experimental data

### Mental calculation

In 1995, Fernández et al. (1995) described power increases in the delta band during mental calculation, and they proposed that this increase corresponded to what they called “internal concentration”. These authors later demonstrated that this delta activity is present in many other tasks that require internal concentration.

Harmony et al. (1996) recorded the EEG in normal subjects during two different conditions:

- A complex arithmetic task that the subject had to solve and give the answer to verbally, e.g., (85/5)6.
- A control stimulus with similar physic characteristics to the arithmetic symbols and to which the subject had to say “No”, e.g., (&&/&) &.

The stimuli were presented in a video monitor in random order and with equal probability. The EEG was sampled every 5 ms. For the analyses, EEG segments of 1024 ms prior to the presentation of the stimulus and after the presentation of the stimulus were selected by visual inspection. Only correct responses were considered.

Using a narrow band EEG analysis with a resolution of 0.78 Hz, the power in each frequency was averaged separately for the segments before and after the stimulus for both conditions. Repeated measures of ANOVA were computed considering two factors: stimuli (calculus vs. control) and the change in the EEG after the stimuli in comparison with the EEG before the presentation. The most important result was related to the interaction stimuli X change, that is, if there was a greater change in power during calculus than during the control condition. The results showed that increases in power were observed from 1.56 to 5.46 Hz only during calculus and not during the control condition. The results for the delta frequencies were interpreted as related to internal concentration, when it is necessary to inhibit all interferences. However, the results included not only delta but also theta frequencies. Theta increases have been associated with many processes: encoding and memory retrieval (Burgess and Gruzelier, 1997), activation of working memory (Gevins et al., 1997; Krause et al., 2000; Deiber et al., 2007) and allocation of attention related to target stimuli (Missonnier et al., 2006). Klimesch (2012) considered that theta and upper alpha are associated with top-down control processes in two large storage systems (i.e., working memory and long-term memory).

Using data from the same experiment, Harmony et al. (1999) calculated the distributed sources for all frequencies every 0.78 Hz between 0.78 and 18.72 Hz. Frequency domain variable resolution electromagnetic tomography (FD VARETA) was used. This is a distributed inverse solution, constrained by the Montreal Neurological Institute probabilistic atlas, that estimates the spectra of EEG sources. Using the values of all subjects resulting from the source analysis at each point of the grid (voxel) at each frequency, a repeated measures ANOVA was carried out for the following factors: task (calculus vs. control) and EEG segment (pre- vs. post-stimuli). The results showed significant differences (*p* < 0.001) for the condition and the interaction. In the task effect at 3.9 Hz, the maximum *F*-value was within the left inferior frontal gyrus (Broca’s area), whereas at 5.46 Hz, the maximum *F*-value was within the right prefrontal area. The interaction effects were maximal within the left parietal for 3.12 and 12.48 Hz and within the left parieto-temporal cortex for 3.90 Hz.

The significant differences between the sources for arithmetic and control tasks, observed at 3.9 Hz within Broca’s and left parietotemporal cortices, may be considered an increase in internal concentration during the production of internal speech to solve the arithmetic problem. However, this was not the interpretation given in the paper (Harmony et al., 1999) but a retrospective view to be congruent with other results from the same laboratory that were discussed in the present review, wherein the author decided to modify the original interpretation.

The increased current in the arithmetic condition at 5.46 Hz reached its maximum in the right frontal cortex. This result is congruent with previous observations, in which high amplitude theta rhythms over the frontal lobes have been associated with attentive states. Sasaki et al. (1996) also observed high amplitude 5.5 Hz theta waves in both frontal lobes, especially on the right side during mental calculation.

In another experiment (Harmony et al., 2004), the EEG of 10 normal male young adults was recorded during the performance of three different tasks: mental calculation, verbal working memory (VWM) and spatial working memory (SWM). The stimuli used in the three tasks were the same, only the instructions to the subjects were different. The tasks used in this experiment were designed following the model of Baddeley (1999) for the study of WM. The three tasks shared common processes, such as the activation of the Central Executive (CES), and differed in one or more processes. SWM activates the visuospatial scratch pad, and VWM activates the articulatory or phonological loop. Mental calculation includes VWM processes as well as other processes, such as the assignation of magnitudes to numerical quantities, mental calculation per se, the storage of intermediate results, and the retrieval of arithmetic facts from long-term memory.

The EEG segments were analyzed during two different intervals on each trial: “pre-segments” were selected 1280 ms prior to the presentation of the memory set; “post-segments” were selected 1280 ms prior to the presentation of the probe. The distributed sources were calculated with VARETA. Using the values of all subjects resulting from the source analysis at each point of the grid (voxel) at each frequency, a repeated-measures multivariate ANOVA was carried out for the following factors: task (VWM, SWM, calculus) and EEG segment (pre-stimuli vs. post-stimuli).

From 1.56 to 4.68 Hz, the frontal cortex showed current increases at the post-segment in VWM and calculus. As VWM is a task included in mental calculation, a common activation of the structures related to VWM in these two tasks was expected (Baddeley, 1999). At 1.56 Hz, the SWM task also activated the frontal cortex. Common processes to the three tasks are those related to the CES. Baddeley (1999) proposed that focusing attention is a main function of the CES. In this case, it is possible to suppose that sustained attention to internal processing is one of the common processes. Segment effects for the three tasks were also observed in the whole cortex for frequencies 7.8, 8.58, 9.36, 10.14 and 10.92 Hz, but no interaction effects were observed for these frequencies within the alpha band; this result indicated that the changes at these frequencies are more directly related to the generalized processes involved in the three tasks than to any specific process.

Only frequencies at 2.34, 3.12, 3.90, 5.46 and 6.24 Hz showed significant interactions. In general, for all frequencies, the interaction effect between segments and tasks was observed in the frontal and anterior cingulated regions. These cortices have been related to the inhibition of interferences during attentional processes, suggesting that delta frequencies indicate this type of inhibition. The interaction was also observed at 5.46 and 6.24 Hz in the entire cortex and in the left frontal lobe, respectively. As previously discussed, theta frequencies have been shown to increase during different cognitive processes, and the tasks used involved many different mental operations.

In this experiment, it was also observed that there were specific EEG changes in frequencies within the delta and theta bands; the specific topographies of these changes were related to the different components of WM, according to Baddeley’s model (Hitch, 1978; Baddeley, 1999).

###  Working Memory

The Sternberg paradigm for the study of short memory processes was used for the EEG recording of 10 right-handed young males (Harmony et al., 1996). In this experiment, a memory set of several digits was presented on a video monitor for 1500 ms, and 2 s later, a single digit (probe) was displayed for 300 ms. Two levels of complexity were evaluated: the memory set consisted either of 3 or 5 digits. The stimuli were presented on a video monitor in random order and with equal probability. EEG segments of 1024 ms before and after the presentation of the stimuli were analyzed. Thus, during this last segment, the subject maintained the 3 or 5 digits in memory, thereby activating processes related to internal concentration or attention to internal mentation. Only correct responses were used for the analyses. Using a narrow band EEG analysis with a resolution of 0.78 Hz, the power in each frequency was averaged separately for the segments before and after the stimulus for both conditions. Repeated measures ANOVA were computed considering two factors: stimuli (5 vs. 3) and the change of the EEG after the stimuli in comparison with the EEG before the presentation. The most important result was for the interaction, that is, if there was a greater change in power during the most difficult condition. Power increases were observed from 1.56 to 3.90 Hz; these increases were greater during the difficult compared to the easy condition (Gevins et al., 1997). During the period in which the subjects were maintaining the list of digits in their memory, the currents exhibited higher intensities, primarily in the left orbito-frontal regions, than currents during rest. The results obtained demonstrate that delta activity increases in regions related to the inhibition of interferences during the task.

**Figure 1:**
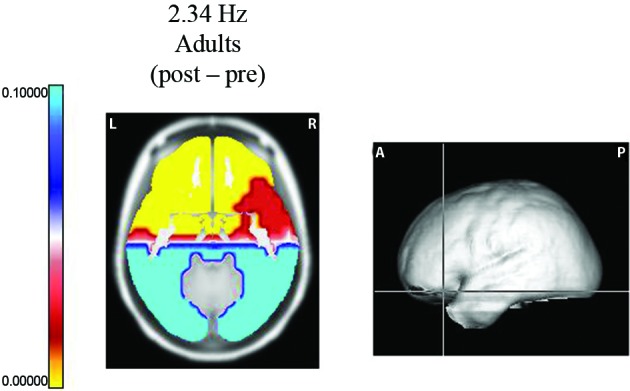
**Probability map for frequency 2.34 Hz**. Sternberg task. Longitudinal slice of the region where the maximum difference was observed between the post segment (when the subject was maintaining the five digits in memory) and the pre EEG segments (before the presentation of the data set). At the right, the levels of the slice on the average brain are shown. Significant increases (yellow color) were observed in the left orbitofrontal region.

Using the Sternberg task, Fernández et al. (2002) studied two samples of subjects: 25 normal, right-handed, 8–10 year old children (14 females) and 15 male, right-handed volunteers (20–26 years old). EEG recordings were made according to the 10/20 system. Each trial in this experiment began with a visual warning stimulus of 300 ms. After an interval of 2 s, a memory set of 5 digits was presented on a video monitor for 1500 ms, and 2 s later, a single digit was displayed for 300 ms. The interval between trials was 3 s. The subject had to respond with one button if the digit was in the memory set and with another button if it was not. Hand use was counterbalanced across the subjects. EEG segments for analysis were obtained before the presentation of the data set (pre-segments) and during the period prior to the presentation of the probe (post-segments). Only correct responses were analyzed. During this last segment, the subject maintained the 3 or 5 digits in memory, thereby activating processes related to internal concentration or attention to internal mentation. In adults, power increases were observed during the post-segments at 1.56, 2.34, 3.90, and 4.68 Hz in the frontal lobes, the anterior regions of the temporal lobes and the anterior cingulate gyri. The most significant change was observed in the prefrontal regions (Figure 1).

However, increases in power during the post-segment were also observed in other frequencies: at 6.24 Hz in frontal regions and at 12.24, 13.26, and 14.04 Hz in large areas of the cortex. Increases at 6.24 Hz are in agreement with previous references (Gevins et al., 1997; Missonnier et al., 2006) as well as the frequencies in high alpha and low beta (Klimesch, 1999; Palva and Palva, 2007; Klimesch, 2012) during WM tasks. Decreases in power were found at 7.02, 7.8, 8.58, 9.36, and 10.14 Hz in the bilateral occipital and temporal lobes; this effect may be related to activation by the visual stimuli.

In contrast, power did not increase in the delta frequencies in children; in this group, increases in power were observed within theta frequencies in the majority of the cortex and decreases in power were observed in the alpha band. The differences between the EEG responses in children and adults, mainly in the delta frequencies in the frontal lobes, which have been related to inhibition, may be related to the well-known fact that children have a deficient inhibition due to an immature frontal lobe.

Changes at 5.46 Hz were of greater intensity in children than in adults. The changes were in the prefrontal and anterior cingulate cortices, which have been related to the attentional role of the CES in working memory. A more pronounced effect in children than in adults may be interpreted as a greater effort for children because these frequencies have been shown to increase with memory load (Gevins et al., 1997).

Important differences between children and adults were also observed at 7.8 Hz in the frontal lobes. Power decreased in adults, while it increased in children. This result is in agreement with the hypothesis of an immature response in children.

### Inhibition

Harmony et al. (2009) performed an experiment using a Go/No-Go task in 15 normal young volunteers (9 females and 6 males, *M* = 26.5 years). The cue letter to prepare the motor response was the letter “O”. When a letter “X” followed the cue letter, the subject was instructed to execute the motor response (Go condition). If a letter different from “X” followed the cue letter “O”, the subject was instructed to inhibit the prepared motor response and to refrain from executing it (No-Go condition).

**Figure 2:**
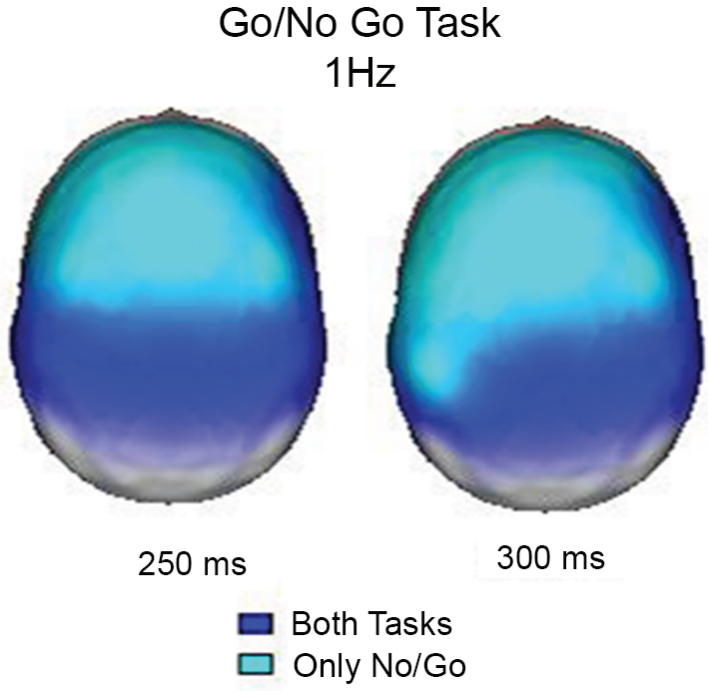
**Surface images of the significant (*p* < 0.01) increases in power at 1 Hz during the Go/NoGo Task**. In both tasks (Go and NoGo) the posterior regions increased power at 250 and 300 ms, however, only during the NoGo task the increase in power was observed in frontal areas.

In this experiment, the event-related EEG responses were analyzed according to Marroquín et al. (2004). Raw EEG signals were passed through a bank of band-pass sinusoidal quadrature filters centered at 1 Hz intervals (from 1 to 50 Hz); each filter had a bandwidth of approximately 1.76 Hz. A power analysis was performed by taking the log-power of each filtered signal and subtracting the pre-stimulus average. In this manner, the time-power-topography maps were obtained for each condition. These maps indicate whether the power increased or decreased during the condition in relation to the pre-stimulus period. Figure 2 shows the maps of increased power at 1 Hz 250 ms and 350 ms after the stimuli, when both conditions coincide in their activation and when the activation was exclusive for the No-Go condition. The results obtained in this study clearly demonstrated that during the inhibition of movement, delta activity increases in the frontal regions; this finding is in agreement with previous knowledge that the frontal regions are particularly important in this process (Vincent et al., 2008). According to Knyazev (2007), there is a reciprocal relationship between alpha and delta activity, and this relationship may reflect an inhibitory control over motivational and emotional drives that is implemented by the prefrontal cortex.

In the Go/No-Go paradigm, it is possible to identify different processes: activation of the visual pathway, letter identification and discrimination processes. Specific processes for the Go condition include the preparation for movement and motor response, whereas the No-Go condition includes the inhibition of the response. Attention is a process common to both conditions because the paradigm mainly explores sustained attention.

Increases in power were observed at 1 Hz in both conditions. In general, this slow frequency is not reported, but it has been shown to be related to the inhibition of non-relevant stimuli in the previous work of our group (Harmony et al., 1996) (e.g., signal matching and decision making (Basar et al., 2001)); these processes are common to both conditions. At this frequency, synchrony increases were observed during 100–300 ms in both conditions, but only in the No-Go condition from 350 to 550 ms, suggesting its participation in the inhibition of the motor response. Increases in power in the theta band were also common to both conditions. Letter identification includes activation of the working memory and access to semantic memory, which may be related to a power increase in the theta range (2–6 Hz) in all regions for both conditions.

Theta power increase has been reported in several situations: during encoding and memory retrieval (Burgess and Gruzelier, 1997; Klimesch, 1999), activation of working memory (Gevins et al., 1997; Krause et al., 2000; Deiber et al., 2007), and allocation of attention.

Supporting our results, Kamarajan et al. (2004) reported that alcoholics showed significant reductions in delta (1.0–3.0 Hz) and theta (3.5–7.0 Hz) power during No-Go trials compared to controls. This reduction was prominent in the frontal region. They concluded that the decreased delta and theta power associated with No-Go processing suggests a deficient inhibitory control and information-processing mechanism in alcoholics.

###  Semantic Processing

A total of 16 control children (8 male) and 18 learning disabled (LD) children (14 male) participated in the experiment. All children were right handed. Pairs of words that were semantically related or unrelated were presented to both LD children and control children with good scholastic achievement. During this presentation, the event-related EEG responses were recorded (Fernández et al., 2012). This task was selected because it requires several language processes and other associated processes, such as attention and working memory. This paradigm has been extensively studied using event-related potentials (Barber et al., 2002; Khateb et al., 2010) and has been considered as a way to study semantic violations (Kutas and Hillyard, 1980; Polich, 1985; Friederici, 2004); to detect these violations, children must comprehend the meaning of what they are reading.

**Figure 3:**
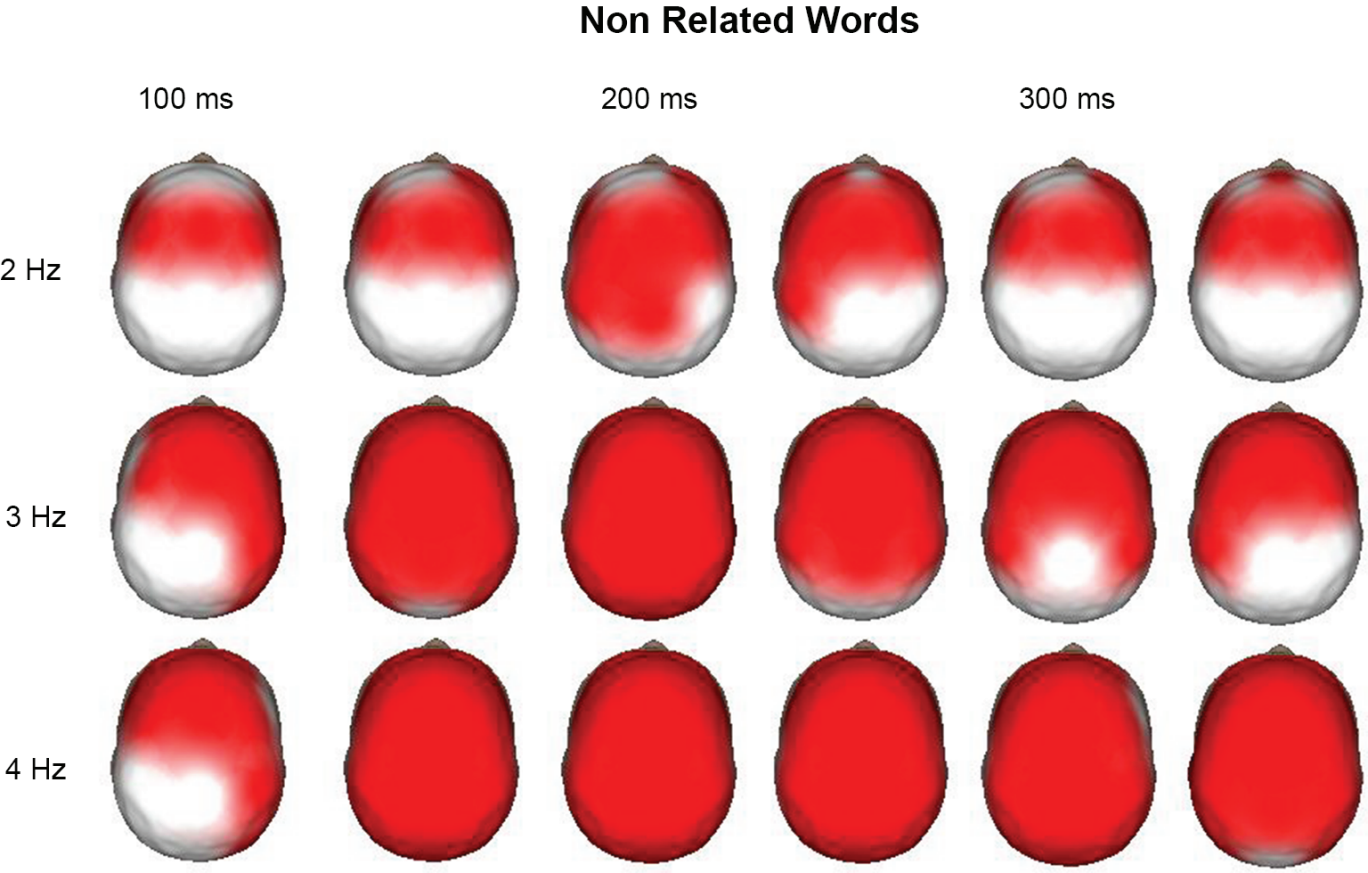
**Surface images of the significant (*p* < 0.01) increases in power at 2, 3, and 4 Hz during the presentation of the non-related words in a semantic task**. Each head is the mean average each 50 ms, from 100 to 350 ms. At 2 Hz the main increases across time are observed in the anterior regions, as well as for 3 and 4 Hz at 100 ms. In these two last frequencies the increase is generalized to all the surface afterwards.

Time-frequency (1–50 Hz) topographic maps were obtained for the increases and decreases of power after the event with respect to the pre-stimulus average values. Figure 3 shows the time-frequency topographic maps, where significant (*p* < 0.01) increases in power with respect to the pre-stimulus were observed from 2 to 4 Hz. It is interesting that for 2 Hz, this increase was observed mainly in anterior regions during the time interval from 100 to 350 ms after the presentation on the nonrelated word. At 3 and 4 Hz, power increases were more generalized; at later points, these increases were observed in all derivations. After the presentation of the word, the child should give a response by pressing different buttons of the mouse according to whether this word was semantically related or nonrelated to a word previously presented; thus, the child should concentrate to give a correct answer and block interferences. It was considered that delta activity at 2 Hz was performing this inhibitory process for the motor processes, whereas activity at 3 and 4 Hz was mainly inhibiting sensory processes.

### Meditation

Important evidence regarding the increase in delta activity during the suppression of interference can be found in some reports involving meditation. For example, a group of Zen meditators exhibited increased delta activity primarily in the medial prefrontal cortex during rest compared to controls (Faber et al., 2008); this increased activity was interpreted as an inhibition of the medial prefrontal cortex, resulting in a reduction of emotional and cognitive engagement, which was described by the Zen meditators as detachment. Subsequently, in searching for evidence of a neuroplasticity effect of continued meditation practice, Tei et al. (2009) measured EEG during no-task eyes-closed resting in a group of Qigong meditators compared with a meditation-naive control group. Significant differences between the groups were only observed in the delta EEG frequency band (1.5–3.5). Regions of the prefrontal cortex and the anterior cingulate cortex exhibited inhibition in the meditators compared to the controls. This inhibition was also observed using functional MRI in the meditators (Hölzel et al., 2007).

Lehmann et al. (2012) studied the connectivity between the different cortical regions in meditators of five meditation traditions. The authors reported that when the meditators went into and out of meditation, significant differences in the connectivities of the meditators across traditions revealed clearly different topographies in the delta frequency. The global reduction in functional interdependence between brain regions in meditation suggests that the interaction between the self-process functions is minimized, leading to the subjective experience of non-involvement, detachment and letting go during meditation as well as an all-oneness and the dissolution of ego borders.

## Conclusions

The aim of the present review was to describe fundamental aspects in relation to the functional significance of delta oscillations in cognitive processing. Evidence suggests that oscillations of the electric field of the brain modulate different networks. This fact may explain the role of delta in many cognitive processes. As we have commented, the wave length of the oscillatory category determines the temporal windows of processing and, indirectly, the size of the neuronal pool involved. Thus, it is possible that during cognitive tasks demanding attention, delta that originates in the frontal cortex may modulate the activity of neuronal networks that are distant from the frontal lobes.

An alternative explanation for the link between delta and cognition has been proposed by Knyazev (2012). He considered that this link is moderated by motivation because it is well known that cognitive performance improves when motivation is high. Our findings have been obtained from subjects that perform adequately in the task, and only correct responses have been considered for the analysis of their EEG. Therefore, we cannot discard the possibility that motivation plays a role during the performance of the task.

The fact that delta activity is characteristic of a state in which interneurons and the thalamocortical inputs are inactive strongly suggests that power increases of delta oscillations during mental tasks have the role of inhibiting all the interferences that may affect the performance of the task.

Another possible mechanism in relation to inhibition for delta oscillations has been proposed by Knyazev and Slobodskaya (2003). They speculated that delta oscillations are linked with the most ancient phylogenetic system of information transmission and that behavioral inhibition is associated with a stronger mechanism of descending inhibition, in which higher systems inhibit the lower. This mechanism was measured by negative correlations between delta, theta and alpha powers. Knyazev (2012) also evaluated the cross-frequency coupling between delta and beta oscillations; increases in the correlation between these two oscillations were present during anxiogenic situations in the orbitofrontal and anterior cingulate cortices.

Thus, delta and beta power were correlated, and this relation appeared to be state dependent, present only in an anxiogenic situation (Knyazev et al., 2006). These data may reflect an interaction between low- and high-frequency oscillations relevant for these oscillatory system situations.

**Future research**: Interactions between different oscillations are important during cognitive processes; however, these interactions have not been explored in many conditions. Our work, up to now, shows deficiencies in this aspect. Future work will focus on these interrelationships. As functional connectivity is a very important aspect in cognitive research, it is important to analyze connectivity in the different frequencies, specifically in the slow bands that have not been studied, during the performance of different mental tasks.

## Conflict of interest statement

The author declares that the research was conducted in the absence of any commercial or financial relationships that could be construed as a potential conflict of interest.
